# Association of *SCN1A* Polymorphisms rs3812718 and rs2298771 with Epilepsy

**DOI:** 10.3390/genes15091224

**Published:** 2024-09-19

**Authors:** Martha-Spyridoula Katsarou, Anna Siatouni, Danae Tsikrika, Elena Kokkiou, Maria Stefanatou, Anastasia Verentzioti, Athanasia Alexoudi, Stylianos Gatzonis, Nikolaos Drakoulis, Maria Papasavva

**Affiliations:** 1 Research Group of Clinical Pharmacology and Pharmacogenomics, Faculty of Pharmacy, School of Health Sciences, National and Kapodistrian University of Athens, 15771 Athens, Greecedrakoulis@pharm.uoa.gr (N.D.); 2 Epilepsy Unit, First Department of Neurosurgery, Evangelismos Hospital, School of Medicine, National and Kapodistrian University of Athens, 15771 Athens, Greecesgatzon@med.uoa.gr (S.G.); 3 Department of Pharmacy, School of Health Sciences, Frederick University, 1036 Nicosia, Cyprus

**Keywords:** neurological disorders, generalized onset seizures, focal to bilateral tonic–clonic seizures, focal onset seizures, single nucleotide variants (SNVs), pharmacogenetics

## Abstract

**Background/Objectives:** Epilepsy is a brain disease with both environmental and genetic inputs. Ion channel dysfunction seems to be of great significance for abnormal neuronal behavior during epileptic seizures. Within neurons, the voltage-gated sodium channels are crucial proteins contributing to the initiation and propagation of action potentials. The voltage-gated sodium channel α subunit 1 (*SCN1A*) gene encodes for the α subunit of a voltage-gated ion channel. The aim of the study was to investigate the relation of two common *SCN1A* variants, i.e., rs3812718 and rs2298771, with distinct epileptic phenotypes in a South-Eastern European population. **Methods:** DNA was extracted from 214 unrelated participants with focal onset, focal to bilateral tonic–clonic, or generalized onset epileptic seizures and genotyped using real-time PCR (LightSNiP assays) followed by melting curve analysis. Statistical analysis of the results was performed using IBM SPSS Statistics software (version 29.0 for Windows). **Results:** Genotype frequency distribution analysis indicated an association for the A-allele-containing genotypes of both rs3812718 and rs2298771 polymorphisms of *SCN1A* with generalized onset seizures and focal to bilateral tonic–clonic seizures versus focal onset seizures. **Conclusions:** Consequently, the study provides evidence that supports a potential association of the investigated *SCN1A* polymorphisms with distinct seizure subtype susceptibility in South-Eastern Europeans.

## 1. Introduction

Epilepsy represents a significant public health concern that affects around 70 million people worldwide [1,2]. It is characterized by abnormal excessive or synchronized neuronal activity in the brain generating transient clinical signs or symptoms termed epileptic seizures [3]. This brain disease is diagnosed when one of the following conditions occur: at least two unprovoked or reflex seizures arising greater than 24 h apart; a single unprovoked or reflex seizure and at least 60% risk of having further seizures over the following ten years; a diagnosis of an epilepsy syndrome [4]. The classification of seizures and epilepsy was developed by the International League Against Epilepsy (ILAE) in 1964 with subsequent updated revisions [5,6,7,8,9,10,11]. According to the latest update, the classification is made at three distinct levels including seizure type, epilepsy type, and epileptic syndrome, and the identification of the etiology and comorbidities at each level is highlighted. The etiology falls into six diverse categories: structural, genetic, infectious, metabolic, immune, and unknown [10].

The notion of epileptogenesis represents the development of the epilepsy state. It comprises a cascade of events that lead to the transformation of the normal brain to one that is susceptible to seizures. This process involves a heightened excitability of specific groups of neurons, making them prone to abnormal discharges [12]. An imbalance between excitation and inhibition within a neuronal network seems to result in epileptogenesis. Epileptogenic networks are broadly distributed in the case of generalized epilepsies, including, bilaterally, thalamocortical structures. Conversely, in focal epilepsies, networks include neuronal circuits limited to one cerebral hemisphere, usually limbic or neocortical [2]. Genetic polymorphisms, in combination with epigenetic alteration and environmental components, serve as key factors in the reconfiguration of neuronal circuits and, thus, the emergence of epilepsy. The subsequent brain adaptations can alter the normal excitatory–inhibitory balance, resulting in the occurrence of a seizure in distinct brain areas and its propagation to other synaptically linked regions, leading to an intensification in the severity of the condition [13]. A genetic component seems to be more prominent in the majority of generalized epilepsies, whereas focal epilepsies have been primarily attributed to structural abnormalities in the brain, especially in cases of drug-resistant epilepsy. The underlying mechanisms through which structural abnormalities provoke seizure activity are not yet completely understood. Nevertheless, focal epilepsies can also have a genetic component [2].

The voltage-gated sodium channel α subunit 1 (*SCN1A*) gene is in chromosome 2 (2q24.3), contains 26 exons and spans 91,480 bp of DNA. *SCN1A* encodes for the α subunit of a voltage-gated ion channel (NaV1.1). Within neurons, the voltage-gated sodium channels are crucial proteins contributing to the initiation and propagation of action potentials through alteration of the permeability of the cellular membrane to sodium ions and, thus, enabling the flux of sodium down an electrochemical gradient to the sodium equilibrium potential [14,15]. Compelling evidence indicates an important role for the *SCN1A* gene in the pathogenesis of epilepsy. In particular, pathogenic variants in the *SCN1A* gene have been correlated with a wide spectrum of epilepsy phenotypes, including genetic epilepsy with febrile seizures, Dravet Syndrome, developmental and epileptic encephalopathies, and epilepsy of infancy with migrating focal seizures [16]. Moreover, polymorphisms in the *SCN1A* have been associated with the responsiveness of antiepileptic drugs (AEDs) [17]. NaV channels are molecular targets for various AEDs including carbamazepine, oxcarbazepine, lamotrigine, phenytoin, and valproic acid [18]. These AEDs exert their antiseizure activity by preventing the diffusion of sodium ions through sodium ion channels during action potential propagation. *SCN1A* polymorphisms have been suggested to be implicated in sodium channel gating rendering them less sensitive to sodium-channel blockers [19].

Considering the undoubted association of the *SCN1A* gene with epileptogenesis, this study aimed to explore the potential association of two of the most common single nucleotide polymorphisms (SNPs) in the *SCN1A*, namely rs3812718 (IVS5N + 5G > A) and rs2298771 (c.3184A > G/p.Thr1067Ala), with the susceptibility of developing distinct epileptic phenotypes and AED responsiveness in a South-Eastern European Caucasian population.

## 2. Materials and Methods

### 2.1. Study Population

The population of the current study consisted of 214 non-related patients diagnosed with epilepsy residing in the geographical area of Greece. All subjects were recruited from Evangelismos General Hospital, Athens, Greece. The diagnosis of epilepsy was made according to the International League Against Epilepsy (ILAE) guidelines by experienced neurologists [6]. The inclusion criteria for the current study were: age ≥ 18 years; clinically diagnosed with focal onset, focal to bilateral tonic–clonic, or generalized onset epileptic seizures in accordance with the ILAE criteria; documented clinical response to the administered AEDs; neuroimaging (magnetic resonance imaging, (MRI)) to exclude structural brain abnormalities or other conditions that could cause epilepsy or contribute to the patients’ symptoms; and South-Eastern European origin. Detailed demographic and clinical information for each patient were obtained via predesigned questionnaires. Information collected including sex, age, age of epilepsy onset, familial epilepsy history, medical and neurological background, seizure typology, disease classification, seizure frequency and duration, electroencephalogram results, imaging findings, and the prescribed antiepileptic drug (AED) regimen were documented. Each subsequent follow-up appointment detailed the dosage of AEDs utilized in either monotherapy or polytherapy, patient adherence, response to AED therapy, and any observed adverse effects. The study protocol was reviewed and approved by the Ethics Committee of Evangelismos General Hospital (307/23-6-2020) and conducted according to the principles outlined in the Declaration of Helsinki. All study participants provided a written informed consent.

### 2.2. DNA Extraction and Genotyping

A peripheral blood sample of around 15 mL was gathered from each participant and stored at −80 °C. A commercial nucleic acid isolation kit was used for the DNA extraction (InnuPREP Blood DNA Midi Kit, Analytik Jena AG, Jena, Germany), according to the manufacturer’s protocol. DNA samples were stored at −20 °C until further analysis. Genotyping of the two examined *SCN1A* variants, i.e., rs3812718 and rs2298771, was conducted using a real-time polymerase chain reaction (LightCycler^®^ 480; Roche, Basel, Switzerland) with simple probes used for each SNP (LightSNiP Assays; TIBMOLBIOL, Berlin, Germany), according to the manufacturers’ instructions. Melting curve analysis was subsequently performed to identify homozygosity for the wild-type or variant alleles and heterozygosity.

### 2.3. Statistical Analysis

Chi-square (χ^2^) (Pearson or Fischer’s exact) tests were used to examine genotypic and allelic frequency distribution differences among epilepsy subgroups. To unravel the correlation of the selected *SCN1A* variants with distinct subtypes of seizures (i.e., focal onset seizures only, focal to bilateral tonic–clonic seizures, and generalized onset seizures) and drug resistance, crude odds ratios (OR) with their corresponding 95% confidence intervals (95% CI) were calculated under five inheritance models: co-dominant, dominant, recessive, over-dominant genotypic, and allelic. Furthermore, logistic regression analysis was used to perform adjustment for potential confounding factors, i.e., sex. All statistical tests were two-tailed. *p*-values less than 0.05 were considered statistically significant. Statistical analysis was conducted using IBM SPSS Statistics software (version 29.0 for Windows).

## 3. Results

The demographic data and clinical characteristics of the 214 enrolled epilepsy patients are summarized in Table 1. The study population consisted of 106 male and 108 female epilepsy patients aged between 18 to 80 years with South-Eastern European Caucasian (SEC) origin. Drug-resistant epilepsy was reported in 85 patients (39.7%). Of the total study participants, 50 (23.4%) patients were reported having generalized onset seizures, 50 (23.4%) patients were reported as having focal to bilateral tonic–clonic seizures, and 114 (53.2%) patients were reported as having focal onset seizures only.

The genotype frequency distribution analysis for the *SCN1A* rs3812718 and rs2298771 variants differed significantly between patients with generalized onset and focal onset to bilateral tonic–clonic versus focal onset seizures. In particular, homozygosity for the more common G allele of the rs3812718 variant (G/G vs. G/A + A/A: OR (95% CI) = 0.494 (0.248–0.984), *p* = 0.043) and the less common G allele of the rs2298771 variant (G/G vs. G/A + A/A: OR (95% CI) = 0.409 (0.171–0.974), *p* = 0.039) were significantly more prevalent in patients with focal onset seizures compared to patients with generalized onset and focal onset to bilateral tonic–clonic seizures (Table 2). Furthermore, allelic frequency distribution analysis indicated a statistically higher prevalence of the rs3812718 G allele in patients with focal onset seizures compared to those with generalized onset and focal onset to bilateral tonic–clonic seizures (G vs. A: OR (95% CI) = 0.661 (0.451–0.970), *p* = 0.034). Regarding the allelic frequency distribution analysis for the rs2298771 variant, a trend for higher occurrence of the G allele was observed in patients with focal onset seizures, though it was marginally non-statistically significant (A vs. G: OR (95% CI) = 1.450 (0.977–2.153), *p* = 0.065) (Table 2). Subgroup analysis between focal onset seizures versus focal to bilateral tonic–clonic seizures (Table 3) and focal onset versus generalized onset seizures (Table 4) did not reveal significant differences, although a trend of association was indicated for the rs3812718 G allele with focal onset seizures (*p* = 0.073) (Table 3). No statistically significant differences of the genotype and allele frequency distributions were found between focal onset and focal to bilateral tonic–clonic versus generalized onset seizures (*p* > 0.05) (Table 5).

The genotype and allelic frequency distribution analysis for the rs2298771 variant did not reveal any significant correlation with drug-resistance (*p* = 0.716 and *p* = 0.691 for A-containing genotype and allelic frequencies, respectively). Similarly, the distribution of the *SCN1A* rs3812718 variant genotypes and alleles did not differ significantly between drug-resistant and non-resistant epilepsy patients (*p* = 0.699, and *p* = 0.575 for A-containing genotype and allelic frequencies, respectively) (Table 6).

## 4. Discussion

The current study investigated the association of two common polymorphisms in the *SCN1A* gene, namely rs3812718 (IVS5N + 5G > A) and rs2298771 (c.3184A > G/p.Thr1067Ala), with the susceptibility of developing diverse epileptic phenotypes and features in a South-Eastern European population residing in Greece. According to the genotypic and allelic frequency distribution analysis, a trend of association for both genetic variants with the occurrence and development of generalized onset and focal to bilateral tonic–clonic seizures was revealed in the study cohort, with the A-containing genotypes (GA + AA) being more prevalent in patients with generalized onset and focal to bilateral tonic–clonic seizures compared to patients with focal onset seizures. Consequently, variability in the *SCN1A* gene may serve as a genetic susceptibility factor for the generalization of seizures in South-Eastern Europeans.

Ion channel dysfunction seems to be of great significance for abnormal neuronal behavior during epileptic seizures. Voltage-gated sodium channels in the brain are comprised by a 260 kDa α subunit and four 33–36 kDa β subunits (β1–β4), with the α subunit being the functional subunit that plays a crucial role in the generation and propagation of neuronal action potentials. *SCN1A*, a neuronal voltage-gated sodium channel a1-subunit gene has been extensively investigated [20]. rs3812718 is a widely studied polymorphism in the *SCN1A* gene, a functional intronic polymorphism located in the 5′ splice donor site of a highly conserved, alternatively spliced exon (exon 5N). The polymorphism results in the generation of alternative splicing products, causing altered proportions of exon 5′ transcripts in the neonate and adult brain tissue [21]. In adults with epilepsy, the rs3812718 polymorphism alters the amount of adult and neonatal mRNA transcripts, with the G allele being associated with the expression of both forms, whereas the A allele seems to significantly decrease the 5N form expression compared to the 5A form. In addition, up to half of the transcripts in carriers of the G allele in homozygosity contain the 5N form, while an undetectable level of the 5N form is expressed in carriers of the A allele in homozygosity. The 5N and 5A types differ in three amino acids and this difference might alter the sodium channels’ electrophysiological properties (S4 sensors) [22]. The rs3812718 polymorphism, together with additional genetic polymorphisms of sodium channels, has been proposed to serve as a potential modifying factor for epilepsy susceptibility and response to AED treatment [23]. According to the results of the current study, an association for the A allele of rs3812718 with the generalization of seizures in the South-Eastern European population residing in Greece has been indicated. These results are in accordance with a previous study performed in a population from Thrace, Greece by Angelopoulou et al., which indicated a marginally more prevalent occurrence of the A-containing genotypes in patients with generalized compared to patients with focal onset seizures, with the generalized onset seizure patients almost three times more likely to have an A-containing genotype compared to those with focal onset seizures [24]. A meta-analysis by Tang et al. indicated that the rs3812718 polymorphism is a risk factor of epilepsy with febrile seizures, especially in Caucasians [25]. Furthermore, in a case-control association study of polymorphisms in the voltage-gated sodium channel genes *SCN1A*, *SCN2A*, *SCN3A*, *SCN1B*, and *SCN2B* and epilepsy by Baum et al., rs3812718 appeared to have the strongest association with epilepsy in a population with Han Chinese, Chinese, Indian, and Malay ethnicities, and a subsequent meta-analysis confirmed the association, indicating a protective role for the G allele in epilepsy risk [26].

The rs2298771 polymorphism (c.3184A > G/p.Thr1067Ala), which is located in the *SCN1A* exon region, is an A-to-G variant, resulting in an alanine to threonine substitution [27]. This polymorphism is associated with alteration in the structural and functional features of the Nav channels [28]. The results of the current study indicate an association for the A-containing genotypes with the generalization of seizures in the South-Eastern European population residing in Greece. The correlation between the *SCN1A*-*A3184G* polymorphism and epilepsy risk has been examined in various non-Caucasian populations and, to a lesser extent, in Caucasian populations, yielding inconsistent findings [15]. In accordance with the results of the current study, the study by Baum et al. revealed an association of rs2298771 with symptomatic epilepsy, particularly in Indians. The authors suggested that the observed association may be due to the linkage disequilibrium of rs2298771 with rs3812718 displayed in Indians [26]. In addition, Makoff et al. reported an association for rs2298771 with idiopathic generalized epilepsy, with the minor G allele being less prevalent in patients [29].

Specific limitations should be taken into account when interpreting the results of the current study. First, the relatively small sample size of the study may have insufficient power to reveal associations with small effect size polymorphisms. Likewise, an examination of further polymorphisms in the *SCN1A* gene as well as in additional genes such as *SCN2A*, *SCN3A*, *SCN1B*, and *SCN2B* was not performed, restricting the acquisition of further genetic information. Furthermore, additional confounding factors such as gene–gene or gene–environment interactions were not considered. Last, while the current study focused on genetic associations within a pre-diagnosed cohort of individuals with epilepsy, and, thus, did not require a control group of non-epileptic individuals, future research could consider including a control group to further investigate broader genetic susceptibility to epilepsy. Consequently, to obtain more definite conclusions, larger-scale studies from different ethnic populations considering gene–gene and gene–environment interactions are needed.

## 5. Conclusions

In conclusion, the results of the study provide supportive evidence for a possible association of both rs3812718 and rs2298771 polymorphisms in the *SCN1A* gene with the susceptibility to develop distinct seizure subtypes. The A-allele-containing genotypes for the *SCN1A* rs3812718 and rs2298771 polymorphisms may serve as risk factors for generalized onset and focal to bilateral tonic–clonic seizures in the investigated South-Eastern European population. Regardless of the aforementioned limitations, the results of the study further support previous findings and provide a reference for additional investigations among the South-Eastern European population to transform genetically derived information into clinical applications for precision management of epilepsy.

## Figures and Tables

**Table 1:** Demographic and Clinical Characteristics of the Study Population (*n* = 214).

Age, *n* (%)		
18–25	47	(22.0)
26–30	26	(12.1)
31–35	28	(13.1)
36–40	22	(10.3)
41–50	57	(26.6)
51–60	19	(8.9)
61–70	10	(4.7)
71–80	5	(2.3)
Sex, *n* (%)		
Male	106	(49.5)
Female	108	(50.5)
Smoking, *n* (%)		
Never	131	(61.2)
Former	23	(10.7)
Ever	60	(28.0)
Seizure subtypes, *n* (%)		
Generalized onset	50	(23.4)
Focal onset	114	(53.2)
Focal onset to bilateral tonic–clonic	50	(23.4)
Drug-resistant, *n* (%)	85	(39.7)

**Table 2:** Genotypic and allelic frequency distribution analysis of the *SCN1A* SNPs between patients with generalized onset seizures and focal to bilateral tonic–clonic seizures versus focal onset seizures only.

	Generalized Onset and Focal to Bilateral Tonic–Clonic Seizures (*n* = 100)		Focal Onset Seizures (*n* = 114)		OR (95% CI)	*p*	OR_adj_ (95% CI) *	*p_adj_* *
	*n*	(%)	*n*	(%)				
**rs3812718**								
G/G	15	(15.0)	30	(26.3)	Reference	-	-	-
G/A	53	(53.0)	58	(50.9)	0.547 (0.266–1.128)	0.100	0.569 (0.274–1.183)	0.131
A/A	32	(32.0)	26	(22.8)	0.406 (0.181–0.911)	**0.027**	0.401 (0.177–0.907)	**0.028**
G/A + A/A	85	(85.0)	84	(73.7)	0.494 (0.248–0.984)	**0.043**	0.514 (0.257–1.029)	0.060
A/A	32	(32.0)	26	(22.8)	Reference	-	-	-
G/A + G/G	68	(68.0)	88	(77.2)	1.593 (0.868–2.921)	0.131	1.538 (0.834–2.834)	0.168
G/A	53	(53.0)	58	(50.9)	Reference	-	-	-
A/A + G/G	47	(47.0)	56	(49.1)	1.089 (0.636–1.864)	0.756	1.090 (0.634–1.874)	0.754
G	83	(41.5)	118	(51.8)	Reference	-	-	-
A	117	(58.5)	110	(48.2)	0.661 (0.451–0.970)	**0.034**	-	-
**rs2298771**								
A/A	42	(42.0)	39	(34.2)	Reference	-	-	-
A/G	50	(50.0)	55	(48.2)	1.185 (0.663–2.116)	0.567	1.122 (0.620–2.029)	0.704
G/G	8	(8.0)	20	(17.5)	2.692 (1.064–6.814)	**0.033**	2.727 (1.071–6.945)	**0.035**
A/G + G/G	58	(58.0)	75	(65.8)	1.393 (0.800–2.424)	0.241	1.344 (0.767–2.354)	0.301
G/G	8	(8.0)	20	(17.5)	Reference	-	-	-
A/G + A/A	92	(92.0)	94	(82.5)	0.409 (0.171–0.974)	**0.039**	0.403 (0.167–0.972)	**0.043**
A/G	50	(50.0)	55	(48.2)	Reference			
G/G + A/A	50	(50.0)	59	(51.8)	1.073 (0.627–1.836)	0.798	1.114 (0.647–1.920)	0.697
A	134	(67.0)	133	(58.3)	Reference	-	-	-
G	66	(33.0)	95	(41.7)	1.450 (0.977–2.153)	0.065	-	-

OR, Odds Ratio; CI, Confidence Interval Bold values indicate statistical significance; * Adjusted for sex.

**Table 3:** Genotypic and allelic frequency distribution analysis of the *SCN1A* SNPs between patients with focal onset seizures only versus focal to bilateral tonic–clonic seizures.

	Focal Onset Seizures (*n* = 114)		Focal to Bilateral Tonic–Clonic Seizures (*n* = 50)		OR (95% CI)	*p*	OR_adj_ (95% CI) *	*p_adj_* *
	*n*	(%)	*n*	(%)				
**rs3812718**								
G/G	30	(26.3)	8	(16.0)	Reference	-	-	-
G/A	58	(50.9)	25	(50.0)	1.616 (0.651–4.016)	0.299	1.595 (0.641–3.972)	0.316
A/A	26	(22.8)	17	(34.0)	2.452 (0.910–6.605)	0.072	2.582 (0.937–7.117)	0.067
G/A + A/A	84	(73.7)	42	(84.0)	1.875 (0.791–4.446)	0.149	1.832 (0.770–4.358)	0.171
A/A	26	(22.8)	17	(34.0)	Reference	-	-	-
G/A + G/G	88	(77.2)	33	(66.0)	0.574 (0.276–1.191)	0.134	0.593 (0.284–1.240)	0.165
G/A	58	(50.9)	25	(50.0)	Reference	-	-	-
A/A + G/G	56	(49.1)	25	(50.0)	1.036 (0.533–2.014)	0.918	1.021 (0.524–1.991)	0.950
G	118	(51.8)	41	(41.0)	Reference	-	-	-
A	110	(48.2)	59	(59.0)	1.544 (0.959–2.484)	0.073	-	-
**rs2298771**								
A/A	39	(34.2)	21	(42.0)	Reference	-	-	-
A/G	55	(48.2)	25	(50.0)	0.844 (0.415–1.718)	0.640	0.891 (0.434–1.830)	0.754
G/G	20	(17.5)	4	(8.0)	0.371 (0.112–1.230)	0.097	0.371 (0.112–1.229)	0.105
A/G + G/G	75	(65.8)	29	(58.0)	0.718 (0.363–1.420)	0.340	0.737 (0.371–1.463)	0.382
G/G	20	(17.5)	4	(8.0)	Reference	-	-	-
A/G + A/A	94	(82.5)	46	(92.0)	2.447 (0.790–7.574)	0.111	2.484 (0.800–7.710)	0.115
A/G	55	(48.2)	25	(50.0)	Reference			
G/G + A/A	59	(51.8)	25	(50.0)	0.932 (0.479–1.813)	0.836	0.901 (0.460–1.762)	0.760
A	133	(58.3)	67	(67.0)	Reference	-	-	-
G	95	(41.7)	33	(33.0)	0.690 (0.421–1.129)	0.139	-	-

OR, Odds Ratio; CI, Confidence Interval; * Adjusted for sex.

**Table 4:** Genotypic and allelic frequency distribution analysis of the *SCN1A* SNPs between patients with focal onset seizures only versus generalized onset seizures.

	Focal Onset Seizures (*n* = 114)		Generalized Onset Seizures (*n* = 50)		OR (95% CI)	*p*	OR_adj_ (95% CI) *	*p_adj_* *
	*n*	(%)	*n*	(%)				
**rs3812718**								
G/G	30	(26.3)	7	(14.0)	Reference	-	-	-
G/A	58	(50.9)	28	(56.0)	2.069 (0.810–5.287)	0.124	2.080 (0.797–5.427)	0.135
A/A	26	(22.8)	15	(30.0)	2.473 (0.874–6.992)	0.083	2.449 (0.862–6.957)	0.093
G/A + A/A	84	(73.7)	43	(86.0)	2.194 (0.891–5.402)	0.082	2.153 (0.867–5.348)	0.099
A/A	26	(22.8)	15	(30.0)	Reference	-	-	-
G/A + G/G	88	(77.2)	35	(70.0)	0.689 (0.327–1.454)	0.327	0.722 (0.339–1.540)	0.400
G/A	58	(50.9)	28	(56.0)	Reference	-	-	-
A/A + G/G	56	(49.1)	22	(44.0)	0.814 (0.417–1.588)	0.545	0.795 (0.404–1.566)	0.508
G	118	(51.8)	42	(42.0)	Reference	-	-	-
A	110	(48.2)	58	(58.0)	1.481 (0.922–2.381)	0.104	-	-
**rs2298771**								
A/A	39	(34.2)	21	(42.0)	Reference	-	-	-
A/G	55	(48.2)	25	(50.0)	0.844 (0.415–1.718)	0.640	0.909 (0.439–1.885)	0.798
G/G	20	(17.5)	4	(8.0)	0.371 (0.112–1.230)	0.097	0.373 (0.113–1.237)	0.107
A/G + G/G	75	(65.8)	29	(58.0)	0.718 (0.363–1.420)	0.340	0.752 (0.377–1.501)	0.419
G/G	20	(17.5)	4	(8.0)	Reference	-	-	-
A/G + A/A	94	(82.5)	46	(92.0)	2.447 (0.790–7.574)	0.111	2.497 (0.798–7.813)	0.116
A/G	55	(48.2)	25	(50.0)	Reference			
G/G + A/A	59	(51.8)	25	(50.0)	0.932 (0.479–1.813)	0.836	0.883 (0.449–1.736)	0.718
A	133	(58.3)	67	(67.0)	Reference	-	-	-
G	95	(41.7)	33	(33.0)	0.690 (0.421–1.129)	0.139	-	-

OR, Odds Ratio; CI, Confidence Interval; * Adjusted for sex.

**Table 5:** Genotypic and allelic frequency distribution analysis of the *SCN1A* SNPs between patients with focal onset versus generalized onset of seizures.

	Focal Onset Only and Focal to Bilateral Tonic–Clonic Seizures (*n* = 164)		Generalized Onset Seizures (*n* = 50)		OR (95% CI)	*p*	OR_adj_ (95% CI) *	*p_adj_* *
	*n*	(%)	*n*	(%)				
**rs3812718**								
G/G	38	(23.2)	7	(14.0)	Reference	-	-	-
G/A	83	(50.6)	28	(56.0)	1.831 (0.735–4.563)	0.190	1.731 (0.685–4.375)	0.246
A/A	43	(26.2)	15	(30.0)	1.894 (0.698–5.135)	0.206	1.861 (0.680–5.094)	0.227
G/A + A/A	126	(76.8)	43	(86.0)	1.853 (0.770–4.455)	0.164	1.757 (0.726–4.253)	0.211
A/A	43	(26.2)	15	(30.0)	Reference	-	-	-
G/A + G/G	121	(73.8)	35	(70.0)	0.829 (0.413–1.666)	0.599	0.872 (0.431–1.764)	0.702
G/A	83	(50.6)	28	(56.0)	Reference	-	-	-
A/A + G/G	81	(49.4)	22	(44.0)	0.805 (0.426–1.522)	0.504	0.802 (0.422–1.524)	0.501
G	159	(48.5)	42	(42.0)	Reference	-	-	-
A	169	(51.5)	58	(58.0)	1.299 (0.827–2.042)	0.256	-	-
**rs2298771**								
A/A	60	(36.6)	21	(42.0)	Reference	-	-	-
A/G	80	(48.8)	25	(50.0)	0.893 (0.457–1.744)	0.740	0.946 (0.479–1.868)	0.873
G/G	24	(14.6)	4	(8.0)	0.476 (0.148–1.533)	0.207	0.480 (0.149–1.551)	0.220
A/G + G/G	104	(63.4)	29	(58.0)	0.797 (0.418–1.519)	0.490	0.834 (0.434–1.600)	0.584
G/G	24	(14.6)	4	(8.0)	Reference	-	-	-
A/G + A/A	140	(85.4)	46	(92.0)	1.971 (0.650–5.980)	0.223	1.935 (0.634–5.907)	0.246
A/G	80	(48.8)	25	(50.0)	Reference	-	-	-
G/G + A/A	84	(51.2)	25	(50.0)	0.952 (0.506–1.794)	0.880	0.919 (0.485–1.743)	0.797
A	200	(61.0)	67	(67.0)	Reference	-	-	-
G	128	(39.0)	33	(33.0)	0.770 (0.480–1.234)	0.276	-	-

OR, Odds Ratio; CI, Confidence Interval; * Adjusted for sex.

**Table 6:** Genotypic and allelic frequency distribution analysis of the *SCN1A* SNPs between drug-resistant and non-resistant epilepsy patients.

	Drug-Resistant Epilepsy Patients (*n* = 85)		Non-Resistant Epilepsy Patients (*n* = 129)		OR (95% CI)	*p*	OR_adj_ (95% CI) *	*p_adj_* *
	*n*	(%)	*n*	(%)				
**rs3812718**								
G/G	19	(22.4)	26	(20.2)	Reference	-	-	-
G/A	39	(45.9)	72	(55.8)	1.349 (0.664–2.740)	0.407	1.288 (0.628–2.642)	0.489
A/A	27	(31.8)	31	(24.0)	0.839 (0.383–1.839)	0.661	0.851 (0.385–1.882)	0.691
G/A + A/A	66	(77.6)	103	(79.8)	1.140 (0.585–2.223)	0.699	1.097 (0.560–2.150)	0.786
A/A	27	(31.8)	31	(24.0)	Reference	-	-	-
G/A + G/G	58	(68.2)	98	(76.0)	1.472 (0.800–2.707)	0.213	1.533 (0.828–2.839)	0.174
G/A	39	(45.9)	72	(55.8)	Reference	-	-	-
A/A + G/G	46	(54.1)	57	(44.2)	0.671 (0.387–1.164)	0.155	0.669 (0.385–1.162)	0.154
G	77	(45.3)	124	(48.1)	Reference	-	-	-
A	93	(54.7)	134	(51.9)	0.895 (0.607–1.319)	0.575	-	-
**rs2298771**								
A/A	35	(41.2)	46	(35.7)	Reference	-	-	-
A/G	38	(44.7)	67	(51.9)	1.342 (0.741–2.427)	0.331	1.379 (0.759–2.506)	0.292
G/G	12	(14.1)	16	(12.4)	1.014 (0.426–2.417)	0.974	1.029 (0.430–2.459)	0.949
A/G + G/G	50	(58.8)	83	(64.3)	1.263 (0.720–2.217)	0.415	1.309 (0.742–2.309)	0.352
G/G	12	(14.1)	16	(12.4)	Reference	-	-	-
A/G + A/A	73	(85.9)	113	(87.6)	1.161 (0.519–2.595)	0.716	1.142 (0.509–2.562)	0.747
A/G	38	(44.7)	67	(51.9)	Reference	-	-	-
G/G + A/A	47	(55.3)	62	(48.1)	0.748 (0.432–1.296)	0.300	0.729 (0.419–1.269)	0.264
A	108	(63.5)	159	(61.6)	Reference	-	-	-
G	62	(36.5)	99	(38.4)	1.085 (0.727–1.619)	0.691	-	-

OR, Odds Ratio; CI, Confidence Interval; * Adjusted for sex.

## Data Availability

Available upon reasonable request.
